# Dental Materials for Oral Microbiota Dysbiosis: An Update

**DOI:** 10.3389/fcimb.2022.900918

**Published:** 2022-06-30

**Authors:** Jieyu Zhu, Wenlin Chu, Jun Luo, Jiaojiao Yang, Libang He, Jiyao Li

**Affiliations:** ^1^ State Key Laboratory of Oral Diseases, Department of Cariology and Endodontics, National Clinical Research Center for Oral Diseases, West China Hospital of Stomatology, Sichuan University, Chengdu, China; ^2^ College of Polymer Science and Engineering, State Key Laboratory of Polymer Materials Engineering, Sichuan University, Chengdu, China

**Keywords:** oral microbiota, dysbiosis, oral biofilms, anti-fouling, antimicrobial biomaterials, dental applications

## Abstract

The balance or dysbiosis of the microbial community is a major factor in maintaining human health or causing disease. The unique microenvironment of the oral cavity provides optimal conditions for colonization and proliferation of microbiota, regulated through complex biological signaling systems and interactions with the host. Once the oral microbiota is out of balance, microorganisms produce virulence factors and metabolites, which will cause dental caries, periodontal disease, etc. Microbial metabolism and host immune response change the local microenvironment in turn and further promote the excessive proliferation of dominant microbes in dysbiosis. As the product of interdisciplinary development of materials science, stomatology, and biomedical engineering, oral biomaterials are playing an increasingly important role in regulating the balance of the oral microbiome and treating oral diseases. In this perspective, we discuss the mechanisms underlying the pathogenesis of oral microbiota dysbiosis and introduce emerging materials focusing on oral microbiota dysbiosis in recent years, including inorganic materials, organic materials, and some biomolecules. In addition, the limitations of the current study and possible research trends are also summarized. It is hoped that this review can provide reference and enlightenment for subsequent research on effective treatment strategies for diseases related to oral microbiota dysbiosis.

## 1 Introduction

The microbiota is involved in the maintenance of host health through multiple pathways. It promotes the maturation of immune cells and the normal development of immune function for immune regulation, acts as a physical barrier to protect the body from foreign pathogens, participates in energy extraction from food, and affects appetite (Wang et al., 2017).

The oral microbiota is an important part of the human microbiota, encompassing over 700 bacterial species, as well as a variety of viruses, fungi, protozoa, and archaea (Deo and Deshmukh, 2019). A healthy individual has 100 to 200+ species of resident bacteria colonized in the oral cavity (Rosier et al., 2018). Fungi are also involved in constituting healthy oral microbiota, while their loads are orders of magnitude lower than bacteria, their size and morphology and synergy with bacteria are crucial in the construction of dental plaque (Diaz et al., 2017). Mark Welch et al. combined sequencing data with spectral fluorescence imaging and revealed that 13 genera are abundant and highly prevalent both in supragingival and subgingival plaque: *Corynebacterium*, *Capnocytophaga*, *Fusobacterium*, *Lepidium*, *Actinomyces*, *Streptococcus*, *Neisseria*, *Haemophilus/Aggregobacteria*, *Porphyromonas*, *Rothella*, *Lautropia*, *Veillonella* and *Prevotella* (Mark Welch et al., 2016).

One of the unique features of the oral cavity compared to the anatomy of other parts of the human body is the presence of teeth. Due to its unique anatomy, the oral cavity contains several distinct ecological niches such as saliva, soft tissue surfaces of the mucosa and hard tissue surfaces of teeth, with different microbial communities (Schwiertz, 2016). The mucous has a constantly renewed physiological process, and the shedding of its aging epithelium is not conducive to the long-term colonization of bacteria (Costalonga and Herzberg, 2014). The salivary microbiota is mainly derived from the shedding of biofilms on the surface of oral tissues, covering 3621 bacterial taxa, of which *Bacteroidetes* (genus *Prevotella*) and *Firmicutes* (genus *Streptococcus* and *Veillonella*) are the main phyla (Keijser et al., 2008). The cheek and palate surfaces have only a single layer of bacteria due to the continuous sloughing of the superficial epithelial layers. However, the tongue surface has multiple layers of biofilm-like bacteria, mainly including *Streptococcus salivarius* (*S. salivarius*), *Rothia mucilaginosa*, and an uncharacterized species of *Eubacterium* (strain FTB41) (Kazor et al., 2003). Significantly, teeth protrude from the mucosal tissue that covers the oral cavity, providing a stable surface for bacterial biofilm formation (Tuominen and Rautava, 2021). According to the location, microbiota on the teeth surface can be divided into two parts: supragingival microbiota (above the gum) and subgingival microbiota (below the gum), which will be described in detail below.

As the most common form of oral microbiota, oral biofilms constitute dynamic, interrelated metabolic networks, whose composition and activity are mainly determined by environment and host (Mclean, 2014). Oral Biofilms are organized communities containing large varieties of microbes embedded in a matrix of extracellular polymeric substances (EPS), whose scaffold is composed of biological macromolecules such as protein, carbohydrate, and nucleic acid (Kuang et al., 2018). The complex microbial network exists interspecies cross-feeding and obtains nutrients, sugars, and amino acids from mucin-containing saliva through the function of glycosidases (Mosaddad et al., 2019). The oral microbiota maintains a healthy state of the microenvironment through multiple pathways. *Veillonella* spp., as one of the main anaerobic bacteria in the oral cavity, is considered beneficial attributes to their abilities that metabolize lactic acid to weaker acids and transfer nitrate $\left({\text{NO}}_{3}^{-}\right)$ to nitrite $\left({\text{NO}}_{2}^{-}\right)$ (Wicaksono et al., 2020). In addition, there are two main pathways for oral microbiota to generate alkali. Some species like *S. salivarius* and *Actinomyces naeslundii* (*A. naeslundii*) metabolize urea by urease enzymes to produce alkali. The other route is the arginine deiminase system (ADS), from which ADS-positive bacteria like *Streptococcus sanguinis* (*S. sanguinis*) metabolize arginine and yield ornithine, ammonia, ATP, and CO_2_ (Liu et al., 2012; Huang et al., 2018). The metabolism of urea and arginine increases local pH, prevents demineralization and promotes remineralization, and also establishes ecological advantages for commensal bacteria and inhibits the growth of various pathogens, thereby maintaining a healthy oral environment (Bowen et al., 2018).

This review addresses the mechanisms underlying the role of the oral microbiota in health and disease states, with a focus on oral diseases caused by microbiota disturbances, including caries, periodontal diseases and peri-implant diseases. On this basis, the emerging materials developed in recent years are reviewed, which are mainly divided into two categories: antifouling materials (covering polymeric agents, biomolecules and metal oxides) and antibacterial materials (covering metals and metal oxides, inorganic nonmetallic materials, organic small molecules, polymers and antimicrobial peptides).

## 2 Oral Microbiota-Related Diseases

### 2.1 Dental Caries

Dental caries, also known as tooth decay, is one of the most prevalent chronic diseases in the world, which can damage both crown and root surface throughout the life cycle, whether in primary or permanent dentition (Selwitz et al., 2007). It is the leading cause of pain and tooth loss in the mouth. As a biofilm-mediated, sugar-driven and multifactorial disease, caries brings about dynamic demineralization and remineralization of dental hard tissue (Pitts et al., 2017). The etiology of dental caries has developed over the centuries, and the involvement of microbes has been acknowledged as early as the late 1800s (Russell, 2009). What can be determined is that the dynamics of carious lesions depend on the availability of fermentable sugars, microbiota, host, and other environmental conditions. However, the specific role of microorganisms in the development and progression of dental caries remains to be further understood.

#### 2.1.1 Supragingival Microbiota

The anatomy of the oral cavity is exceptional compared to that of other human body sites. A unique feature is hard tissue, i.e., teeth that protrude through the mucosa covering a major part of the oral cavity. Teeth provide non-shedding surfaces for distinct bacterial biofilm formation, whereas mucosal surfaces are continuously renewing and older epithelial layers are shedding from the surface, presenting challenges to permanent bacterial colonization (Sedghi et al., 2021). Peculiarly, there is an acquired pellicle covering the teeth surface, which is composed of lipids, proteins, glycolipids, and glycoproteins (Chawhuaveang et al., 2021). Acquired pellicle can protect teeth enamel from acid attack, but also regulates the further attachment of bacteria and promotes the development of biofilm (Thomas et al., 2021).

The structured microbiota is embedded in the EPS matrix consisting of proteins, polysaccharides, lipids, nucleic acids, and other biomolecules and firmly attached to the substrate surface to form biofilms. The physicochemical properties of EPS are critical for the biochemical action of biofilms, including mechanical stability, signal transmission, gene swapping, and antimicrobial tolerance (Karygianni et al., 2020). The initially formed EPS matrix promotes microbial colonization and aggregation, and as the matrix further expands, the EPS wraps around bacterial cells, providing a supportive framework for the development of microscopic colonies (Flemming et al., 2016). The extracellular matrix protects biofilms from mechanical removal and antibacterial agent and creates localized regions of low pH by inhibiting the buffering capacity of saliva, which can facilitate intensive localized acidification and teeth demineralization (Valm, 2019).

The primary initial colonizing bacteria are *Streptococcus*, followed closely by gram-positive bacilli, particularly *Actinomyces* spp. Subsequently, other cocci and bacilli gradually attached to the foregoing gram-positive biofilm (Larsen and Fiehn, 2017). Among them, *Fusobacterium nucleatum* (*F. nucleatum*) plays an essential role in the maturation of biofilms by co-aggregating with the initial bacteria and succeeding gram-negative and motile bacteria, such as *Bacteroidetes* and *Spirochaetes* (Benitez-Paez et al., 2014). Eventually, the cariogenic microbiota is dominated by thriving acidogenic and aciduric microorganisms, including mutans and non-mutans *Streptococcus*, *Actinomyces*, *Bifidobacterium*, *Lactobacillus*, and *Scardovia* spp., whose further synergistic effect will promote EPS generate and microenvironment acidification (Lamont et al., 2018).

Recent advances based on DNA and RNA techniques have further shed light on the microbiota associated with caries. In carious lesions whether in enamel or dentin, the supragingival microbiota dramatically decreased from 500-700 species to 100-200 species-level phylotypes (Simon-Soro and Mira, 2015). The bacteria involved in enamel caries were mainly *Veillonella*, *Rothia*, and *Leptotrichia*, while the bacteria involved in dentin caries were mainly *S. sanguinis*, *Atopobium*, *Schlegelella*, *Pseudoromibacter*, and *Lactobacilli* (Vanaki, 2020). *Streptococcus mutans* (*S. mutans*) and *Lactobacillus* are closely related to dental caries, which can ferment sucrose to polysaccharides and produce lactic and ATP (Zeng and Burne, 2016; Tanner et al., 2018). Some other common cariogenic bacteria exhibit the high potential of sugar decomposition and acid production, including *Corynebacterium*, *Granulicatella*, *Propionibacterium*, and certain strains of *Leptotrichia* (Lamont et al., 2018). And the lactic can be utilized as a carbon source for *Veillonellae*, one of the aciduric species (Chalmers et al., 2008). Besides, *Candida albicans* (*C. albicans*) interact with glucosyltransferases produced by *S. mutans*, enhancing the virulence of the biofilm matrix, which plays a crucial role in early childhood caries (Koo et al., 2018).

#### 2.1.2 Diet and Microbiota

Frequent intake of carbohydrates plays an important role in altering the oral microbiota. Tanner et al. suggested that caries is the result of an imbalance between acid-producing and acid-tolerant bacteria, which is closely related to a frequent diet containing sugar or carbohydrates (Tanner et al., 2018). When sugar intake is low and infrequent, the microbiota on the teeth can remain stable and the small amount of acid production can be easily neutralized by saliva, protecting the teeth from acid erosion and demineralization (Takahashi and Nyvad, 2011). Overexposure to fermentable carbohydrates facilitates the production of EPS and acidic metabolites, as well as the collection of acidogenic and aciduric microorganisms, thus driving the conversion to pathogenic microbiota (Bowen et al., 2018). Microbes will be embedded in the biofilm matrix when carbohydrates are ingested frequently. As a result, local pH is lowered by continuous acid production that avoids saliva buffer, thereby inducing the mineral balance towards demineralization (Takahashi and Nyvad, 2011).

Ecological perspectives for microbiota dysbiosis in dental caries contain 3 reversible stages (Takahashi and Nyvad, 2011). The healthy state’s microbiota on the enamel surface consists mainly of non-mutans *Streptococci* and *Actinomyces*, with mild and uncommon acid production. When demineralization/remineralization is in equilibrium or the balance is tilted towards mineral gain, it is in a dynamic stability stage. When frequent carbohydrate supplies lead to a prolonged acidic environment, acid production and acidity of non-mutans bacteria are adaptively enhanced, and more aciduric strains selectively increase. Therefore, the demineralization/remineralization balance is induced to shift towards mineral loss and promotes caries development, which is in an acidogenic stage. The prolonged acidic condition further induces acidic selection of aciduric and acidogenic bacteria to become dominant bacteria, including *mutans Streptococci* and *Lactobacilli* as well as aciduric strains of non-mutans *Streptococci*, *Actinomyces*, Bifidobacteria, and yeasts, which is called an aciduric stage.

Compared with glucose, fructose, and starch, sucrose has strong cariogenic potential due to its fermentability and can be used as a substrate for glucosyltransferase of *S. mutans* to synthesize EPS and intracellular polysaccharides (IPS) (Paes Leme et al., 2006). EPS boost bacterial adhesion on tooth surfaces, causing structural and chemical changes of the biofilm matrix, which makes it more difficult to remove biofilm (Liu et al., 2018b). In addition, IPS reduces pH during nutrient deprivation, leading to the selective proliferation of cariogenic microbiota (Costa Oliveira et al., 2021).

Dental caries is an event of microbiota dysbiosis, and diet plays a key role by providing a highly structured and localized acidic microenvironment, promoting caries development through demineralization that conversely shapes the constitution and bioactivity of microbiota. Apparently, challenges existing in controlling cariogenic biofilms mainly include the following aspects. First, the cariogenic microorganisms entangled in the EPS-rich biofilm matrix are protected by the matrix, making it hard to combat or eliminate. Second, EPS generate an extremely acidic microenvironment, promoting the proliferation of cariogenic microbiota and reducing the therapeutic efficacy of drugs. Lastly, because of the rapid refresh effect caused by oral activity and saliva scouring, topical medications barely sustained on biofilms.

### 2.2 Periodontitis

Unlike infections caused by a single microbial pathogen, periodontitis is triggered by the synergy of multiple microbial communities rather than by specific microorganisms. Arguably, periodontitis is not an infectious disease, but a dysbiosis disease, relating to changes in species abundance in the microbiota and the impact of such changes on health (Lamont et al., 2018). In addition, periodontal dysbiosis is in connection with the disruption of tissue homeostasis, largely due to microbial subversion of local immune response (Hajishengallis, 2015). As the disease progresses, further periodontal tissue destruction will eventually lead to loosening and even loss of teeth, directly affecting chewing or speaking function as well as aesthetics, reducing the patient’s quality of life (Pihlstrom et al., 2005).

#### 2.2.1 Subgingival Microbiota

Characteristics of the local environment determine the properties of relevant microbiota. Matching the constant renewal of gingival epithelial cells, the corresponding microbiota develops more rapidly and is less complex than that on the tooth surface (Hajishengallis and Lamont, 2021). Furthermore, to cope with loss upon host cell death, plenty of colonizing bacteria in the junctional epithelium invade tissue and internalize within the epithelium, where they are protected from host immune molecules (Yilmaz et al., 2006; Lee et al., 2020). The subgingival microbiota in health includes gram-positive bacteria and a few numerically abundant gram-negative bacteria, spatially arranged in organized associations and interacting in a physical and metabolic way (Curtis et al., 2020). Among them, gram-positive cocci and rod cells predominated in number during early colonization (Listgarten, 1976). *Actinomyces* spp. can co-aggregate with other bacteria such as *Streptococcus* in initiate colonization stage to construct the skeleton of dental plaque biofilms (Kolenbrander et al., 2006). Notably, there are still health-related species in periodontitis and vice versa, further confirming that periodontal disease is caused by a dysbiosis rather than a single pathogen (Curtis et al., 2020).

Compared with healthy individuals, the total bacterial count of the subgingival microbiota in periodontitis individuals was similar. However, the predominant bacterial species in the subgingival microbiota of the two subjects have a significant difference. The subgingival microbiota in health has higher proportions of *Streptococcus* species, suggesting it is the main component of the health subgingival microbial community. Nevertheless, periodontitis had a higher proportion of obligate anaerobic bacteria in the subgingival microbiota, especially Porphyromonas *gingivalis* (*P. gingivalis*), *Tannerella forsythia* (*T. forsythia*), and *Eubacterium saphenum* (*E. saphenum*) (Abiko et al., 2010). Socransky and his team utilized whole genomic DNA probes and checkerboard DNA-DNA hybridization to distinguish the periodontal microbiotas and create a color-coded system to characterize them. Among them, the “red complex” group consisting of *P. gingivalis*, *T. forsythia*, and *Treponema denticola* (*T. denticola*) is the most closely related to periodontal disease, increasing in number with the depth of periodontal pocket (Socransky et al., 1998; Mineoka et al., 2008). The bridging orange-complex species, i.e., *F. nucleatum* and *Prevotella* spp., and late red-complex colonizers, take longer to mature than fast thriving yellow-complex species in the early colonization such as *Streptococcus* spp. (Teles et al., 2013).

It is obvious that the dysbiosis of the microbiota causes differences in metabolic pathways and functions. Elevated levels of bacterial motility proteins and flagellar assembly may imply an increased invasive capacity of pathogenic bacteria in periodontitis (Cai et al., 2021). Studies have shown that bacterial phenolic acid metabolites, especially phenylacetate and volatile sulfur compounds were positively associated with periodontal exploration depth (Liebsch et al., 2019; Abdullah et al., 2020). In addition, valine, phenylalanine, isoleucine, tyrosine, and butyrate were significantly upregulated in periodontitis subjects, while lactate, pyruvate, and N-acetyl were the most strongly expressed in healthy subjects (Romano et al., 2018).

Co-infection can enhance adhesion and invasion of the red complex to gingival epithelial cells (Li et al., 2015). Synergistic community interaction provides a platform for comprehensive regulation of actions, including obtaining nutrient acquisition, expressing genes, and swapping DNA. It is now well established that, the pathogenicity of periodontal pathogens only becomes meaningful under the interaction of synergistic microbial communities, determining the nature and function of the whole microbiota (Hajishengallis and Lamont, 2012).

#### 2.2.2 Host Immune Defense

Although the predominant colonization of certain bacteria is considered to be closely associated with periodontal disease, they have also been detected in a healthy state. Therefore, it cannot be arbitrarily assumed that these bacteria are the sole cause of periodontal disease, as their pathogenic process requires the evolution from a healthy, organized microbiota to a dysbiotic microbiota, which ultimately promotes inflammation and tissue destruction of the periodontal tissue. It is now widely accepted that periodontitis is an inflammatory disease destructing periodontal soft and hard tissues. Microbiota dysbiosis is an initiating factor of local inflammation, while hyperactivation of the host immune system is the direct factor that stimulates osteoclast activity and causes alveolar bone resorption (Pan et al., 2019).

Under physiological conditions, the immune system does not mount a severe inflammatory response during immune monitoring and tolerance of the microbiota (Graves et al., 2019). However, the immune system will overreact in the context of microbiota dysbiosis, contributing to localized inflammatory infiltration. As the dysregulated microbiota continuously stimulates and hurts periodontal tissue, immune cells such as specific T cell subsets, antigen presenting cells, and mononuclear phagocytes are recruited locally. During this process, the interaction of pattern recognition receptors (PRRs) with pathogen-associated molecular patterns (PAMP) expressed by the pathogen microorganisms leads to the secretion of pro-inflammatory cytokines, including interleukin-1 (IL-1), interleukin-6 (IL-6), and tumor necrosis factor (TNF), which has the function of activating lymphocyte and destroying tissue (Graves, 2008; Gu and Han, 2020). Additionally, immune cells secret a cluster of particular cytokines, activating relevant signaling pathways and promoting the differentiation of specific lymphocyte subsets with the participation of IL-1 and IL-6. These lymphocyte subsets in turn secrete specific patterns of cytokines that serve as positive-feedback factors or direct effectors to regulate the immune response as well as osteoclast activity (Pan et al., 2019).

*P. gingivalis* can secret toxic factors like LPS, gingipains, and pili to directly destroy periodontal tissues, and also activate host immune cells to trigger local immune responses and motivate the release of inflammatory mediators, resulting in secondary tissue damage (Jia et al., 2019). As PAMP recognition receptors, toll-like receptors (TLRs) can mediate the host’s innate immune response to *P. gingivalis*, the foundation of acquired immunity, playing a crucial role in the occurrence and development of periodontitis (Nakayama and Ohara, 2017).

As an important factor in periodontal tissue destruction, matrix metalloproteinases have the ability to decompose the extracellular matrix and basement membrane, representing a group of structurally related but genetically distinct enzymes. The expression of matrix metalloproteinases is low in healthy periodontal tissues. However, when interleukin-8 (IL-8) is secreted in response to bacterial biofilms, neutrophils are recruited to sites containing biofilms and secrete matrix metalloproteinases 8, which mainly degrades interstitial collagen (Sorsa et al., 2006). It has been found that *F. nucleatum* may induce the production of matrix metalloproteinase-13, which can degrade collagens of types I, III and IV, as well as fibronectin (Uitto et al., 2003). The activation of matrix metalloproteinases is a combined result of tissue, plasma and bacterial proteinases, combined with the effects of oxidative stress (Cekici et al., 2014).

Recently, some studies have identified T helper 17 (Th17) cells and correlative cytokines such as interleukin-17 (IL-17) have been implicated in the pathogenesis of periodontitis because of the ability to induce osteoclastogenesis (Cheng et al., 2014; Bunte and Beikler, 2019). The study by Cheng et al. showed that *P. gingivalis* and *Actinobacillus actinomycetemcomitans* enhance Th17/IL-17 responses through activating human CD14(+) monocytes (Cheng et al., 2016).

### 2.3 Peri-Implant Diseases

Over the past 50 years, the application of dental implants to improve chewing efficiency and living quality of patients with loss of teeth has become more and more prevalent due to its remarkable biological advantages (Buser et al., 2017). But in the last 30 years, peri-implant infective diseases have emerged, including peri-implant mucositis only involving peri-implant soft tissue and peri-implantitis that also involves peri-implant bone loss (Zitzmann and Berglundh, 2008; Berglundh et al., 2018). Peri-implant diseases cause implant loosening or eventual removal in most cases, placing a huge financial burden on the patient and severely impairing quality of life (Greenstein and Cavallaro, 2014).

Peri-implant diseases and periodontal diseases share similar risk factors, making their clinical outcomes similar. However, recent proteomic and molecular studies have shown a significant difference between peri-implant diseases and periodontal diseases.

#### 2.3.1 Peri-Implant Microbiota

Dental implants provide a colonized surface for microbiota that differs teeth in roughness, surface energy, morphology, and material. In detail, dental implants are made of titanium and/or ceramics, in the shape of a conical screw, have a higher surface roughness and lower surface energy than teeth, so they are more susceptible to bacterial adhesion, and have greater bacterial abundance (Robitaille et al., 2016).

Surface irregular bacterial colonization begins about 30 minutes after the dental implant is placed in the oral tissue (Van winkelhoff et al., 2000). Driven by van der Waals forces, electrostatic and hydrophobic interactions, bacteria approach and finally adhere to the acquired pellicle, thereby establishing irreversible adhesion, followed by up-regulation of bacterial metabolic activity and extensive bacterial colonization of the implant surface (Wassmann et al., 2017). At the whole-microbiome level, the peri-implant microbiota has comparatively low diversity and less variability, which was characterized by 71 species (Ghensi et al., 2020). The healthy peri-implant oral microenvironment is predominantly colonized by *Streptococcus*, which accounts for 45% to 86% of supragingival and subgingival peri-implant microbiota. Besides, *Actinomyces* as well as *Rothia* and *Neisseria* species have also been continually isolated (Quirynen et al., 2005).

Whereas 12 species were enriched in peri-implantitis: *Fretibacterium fastidiosum* (*F. fastidiosum*), *T. forsythia*, *Desulfobulbus* spp. oral taxon 041, *Treponema socranskii*, *Filifactor alocis*, *T. denticola*, *Porphyromonas endodontalis* (*P. endodontalis*), *Treponema maltophilum*, *Pseudoramibacter alactolyticus*, *Treponema lecithinolyticum*, *P. gingivalis*, *F. nucleatum* (Ghensi et al., 2020). Using 16S rRNA sequencing, Schaumann et al investigated the microbial composition of biofilms at different oral sites in individuals with peri-implantitis (Schaumann et al., 2014). The study found that the most abundant submucosal species on implants were *Rothia*, *Streptococcaceae*, and *Porphyromonas*, while the most abundant subgingival bacteria on teeth were *Prevotella*, *Streptococcaceae*, and *TG5.*

A recent study by Shi et al. determined that the richness, diversity, and distribution of microbiota were very similar between peri-implant mucositis and peri-implantitis, both having the core microbiota: *Porphyromonas*, *Fusobacterium*, *Treponema*, *Prevotella*, and *Campylobacter* (Shi et al., 2022). Compared with periodontal diseases, peri-implant diseases are related to higher levels of *Peptococcus*, *Mycoplasma*, *Eubacterium*, *Campylobacter*, *Butyrivibrio*, *S. mutans*, and *Treponema*, and lower levels of *Prevotella*, non-mutans *Streptococcus*, *Lactobacillus*, *Selenomonas*, *Leptotrichia*, *Actinomyces* (Robitaille et al., 2016). Another study showed some interesting results, such as *Selenomonas artemidis*, *Eikenella corrodens*, *Ottowia* sp. HOT894 and *Neisseria meningitidis* appeared to uniquely be relevant to peri-implants inflammation (Schincaglia et al., 2017). Ghensi et al. suggested defining the “peri-implantitis-related complex” of 7 species strongly characterizing peri-implantitis sites: the red complex triad (*P. gingivalis*, *T. forsythia*, *T. denticola*), the *P. endodontalis* and *F. fastidiosum* species, the *Prevotella intermedia*, and *F. nucleatum* species (Ghensi et al., 2020). Among them, *F. nucleatum* is closely associated with peri-implant diseases, especially peri-implant mucositis, and is also a key bacterium in the microbiota associated with periodontal disease.

In addition, observational studies suggested that peri-implantitis was an intricate and multifactorial infection, associated with opportunistic pathogens such as *Staphylococcus aureus* (*S. aureus*) and *Pseudomonas aeruginosa* (*P. aeruginosa*), fungal organisms (*C. albicans*, *Candida boidinii*, *Paelicomyces* spp., *Penicillum* spp., *Rhadotorula laryngis*), and viruses (human cytomegalovirus, Epstein-Barr virus) (Schwarz et al., 2018).

In a word, the peri-implant disease is associated with dysbiosis in the microbiota, some of which may take part in the initiation of disease while others contribute to disease progression.

#### 2.3.2 Host Immune Defense

Peri-implant mucositis is characterized by changes in the composition of the microbiota with an increase in gram-negative microorganisms and activation of local host responses. Microbiota dysbiosis causes the release of chemotactic peptides and cytokines that recruit leukocytes such as neutrophils to peri-implant pockets, thus engulfing and digesting bacteria. However, if the neutrophils degranulate by excessive bacteria, they will release toxic enzymes and damage peri-implant tissue (Petkovic et al., 2010).

Health-associated bacterial biomarkers include chaperonin, iron uptake protein A2, and phosphoenolpyruvate carboxylase. Some biomarkers like ribulose biphosphate carboxylase, succinyl-CoA:3-ketoacid-coenzyme A transferase, and DNA-directed RNA polymerase subunit beta are specific in periodontitis and are also important in peri-implantitis (Baliban et al., 2012). Chemokines (IL-8 and MIP-1α) and proinflammatory cytokines (IL-1β and TNF-α) may serve as markers for monitoring the condition of peri-implant tissues (Petkovic et al., 2010). Immunohistochemical staining showed that IL-1α expression was more prevalent in peri-implant tissues, whereas TNF-α expression was more prevalent in periodontitis tissues (Konittinen et al., 2006).

The pro-inflammatory molecule IL-17, produced by Th17 cells, modulates multiple biological inflammatory effects, including recruiting neutrophils and macrophages and stimulating other pro-inflammatory mechanisms (Ouyang et al., 2008). Mardegan et al. investigated the Th17 (IL-17 and interleukin-23, IL-23) and Treg (transforming growth factor-β, TGF-β) cytokine gene expression levels in healthy and peri-implantitis tissues (Mardegan et al., 2017). A predominant Th17 response and a reduction of Treg response was observed in peri-implantitis tissue compared to healthy tissue, especially arising from up-regulation of IL-23 and down-regulation of TGF-β around the implant.

Mikolai et al investigated early host-microbe interaction based on a peri-implant oral mucosa-biofilm model and obtained profound knowledge (Mikolai et al., 2020). The study showed *P. gingivalis* is capable of attenuating the PI3K-Akt signaling pathway and disrupting cell-cell junctions at gene and protein levels, thereby enhancing bacterial colonization and damaging the epithelial barrier. Furthermore, the release of antimicrobial peptides or mucosa breakdown products and/or the presence of *P. gingivalis* may lead to altered bacterial distribution with an increased proportion of *Veillonella dispar*, deriving lipopolysaccharides to induce TLR4-dependent host cell responses, which can lead to inflammation. Intriguingly, compared to periodontitis, fibroblasts isolated from peri-implantitis had greater production of matrix metalloproteinases (MMP), vascularizing factors, and complement receptor C1q, and less production of metalloproteinase inhibitors and growth factors, which promote collagen synthesis, which may explain the faster and more extensive tissue destruction in peri-implantitis (Belibasakis, 2014).

Duarte et al. used quantitative polymerase chain reaction to assess the gene expression of different inflammatory factors in gingiva from healthy implants and various degrees of peri-implant diseases (Duarte et al., 2009). The study revealed that, concerning inflammatory factors, IL-12 and TNF-α were higher in severe peri-implantitis, followed by initial peri-implantitis and mucositis, while IL-4 was higher in healthy projects, followed by mucositis, severe, and initial peri-implantitis. In consideration of osteoclastogenesis-related factors, RANKL increased with peri-implantitis severity, while OPG mRNA levels were higher in healthy implants, followed by initial, severe peri-implantitis, and mucositis.

## 3 Materials Strategies

### 3.1 Antifouling Materials

Building an early biofilm asks for the absorption of protein to the solid surfaces to construct the salivary acquired pellicles, along with the adherence of initial colonizers to them. Then the subsequent adhering of other oral pathogens to immobilized bacteria, also known as cohesion or coaggregation, leads to maturation of the biofilm (Kolenbrander et al., 2010). The antifouling property of biomaterials, including protein repulsion and bacteria anti-adhesion, can protect surfaces from invasion of early biofilm. In addition, the accumulation of dead pathogens and bio-foulants on oral surfaces or dental materials can be prevented by introducing the antifouling property, which unblocks other biofunctions (Duan et al., 2022). Current antifouling materials are usually polymeric antifouling agents, besides, some biomolecules and special metals also show the antifouling property.

#### 3.1.1 Polymeric Agents

**Figure 1:**
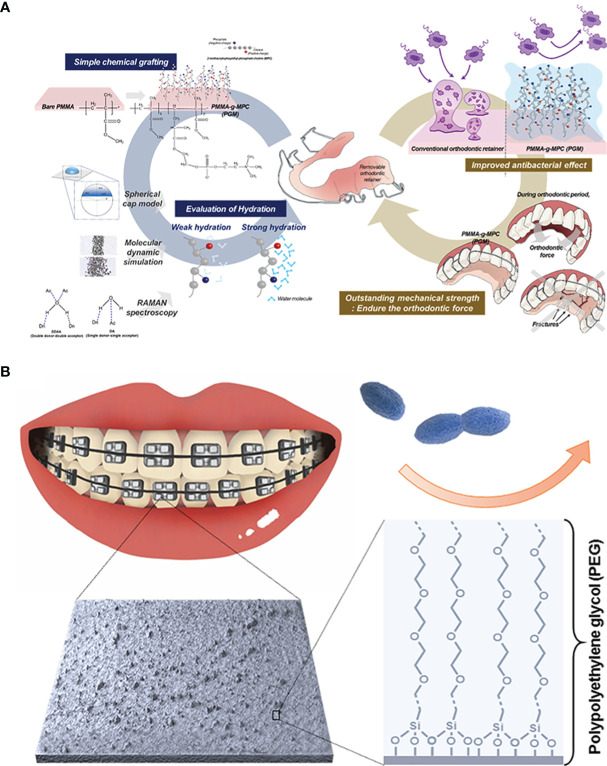
Polymeric anti-fouling strategies: **(A)** zwitterionic antifouling coating grafted onto the PMMA resin for bacterial anti-adhesion; **(B)** hydrophilic PEG-coated stainless steel archwire to achieve antiadhesive property.

An effective intervention for inhibiting the absorption of bacteria and protein on the material surfaces is to reduce the contact area among them. For this purpose, the water barrier effect of hydrophilic materials can play a certain role. In theory, a hydrophilic material usually has strong hydrogen bond interactions with water molecules, which can induce water molecules to bind intensively with material surfaces, leading to the formation of a hydration layer with the shielding effect. The existence of a hydration layer can make it difficult for bacteria and protein to get close to material surfaces, thus achieving a good anti-fouling property (Jin et al., 2022). Currently, polyethylene glycol (PEG) and zwitterionic polymer are the two most widely used hydrophilic anti-fouling materials (Venault et al., 2014; Lee et al., 2019). PEG is a flexible polymer with -CH_2_-CH_2_-O- as the repeated unit, which make it not only has hydrogen bonding with water molecules, but also equip with the steric repulsion effect to prevent the invasion of bacteria and protein. Benefitting from the ion’s solvent effect, zwitterionics has a stronger interaction to form a denser hydration layer. Among all, 2-methacryloyloxyethyl phosphorylcholine (MPC) has been used in the antifouling application due to its optimizable molecular structure (Baggerman et al., 2019). The surface charge can be adjusted by controlling the positive to negative groups ratio. Consequently, the antiadhesion propertied can be tuned. However, materials with the hydrophilic property are not stable to bind with the matrix, which is also the main limitation to develop them as the antifouling coating. Buxadera-Palomero et al. once prepared PEG coatings on the titanium (Ti, still contemplated to be the first choice in dental implant therapy) surface by plasma polymerization (Hwang et al., 2012). Subsequently, they took advantage of the pulsed electrodeposition technology to construct the PEG coating on Ti surfaces (Buxadera-Palomero et al., 2020). Both two means can achieve expected bacteria antiadhesion. But the successful coating by plasma polymerization and electrodeposition depends on extra devices, complicating the whole process. To solve it, the method that PEG or zwitterionics are fixed on the substrate surfaces by chemical grafting has come into view. Choi et al. grafted MPC brushes on the PMMA resins with different grafting efficiencies by the free radical polymerization (Choi et al., 2020). Meanwhile, the hydration and MPC dynamics were evaluated logically and quantitatively by molecular simulation and Raman spectroscope to optimize the antifouling property. The resulting resins proved a nonspecific bacteria antiadhesion behavior aiming at *A. naeslundii*, *S. aureus* and *P. aeruginosa* (**Figure 1A**). Silane chemistry is another available method to graft organic polymers to inorganic substrates. Alkoxysilane of silane coupling agents is reactive to inorganic matter, while organo-functional groups can be compatible with organic matter. Peng et al. prepared silane-ended PEG chain with varied molecular weight and coated it on the tooth stainless steel archwire (Peng et al., 2017). The PEG-coated archwire showed excellent long-term bacteria antiadhesion properties (**Figure 1B**). Coating materials with chemical grafting is only applicable to the modification of dental materials such as implants or resins rather than oral tissue. In view of the abundant existence of Ca^2+^ ions on the tooth surfaces, modifying polymers with groups that can interact with these ions has been an alternative. For example, Hou et al. synthesized a highly hydrophilic diblock copolymer polyethylene glycol-poly (aspartic acid) (PEG-PAsp), where carboxyl groups in the PAsp segments provide binding sites with Ca^2+^ on the enamel surfaces, so that PEG segments on the other side can inhibit *S. mutans* and Stoeptococcus *sanguis* (*S. sanguis*) adhesion on the enamel (Hou et al., 2020). Compared with carboxyl groups, ${\text{PO}}_{4}^{3-}$ groups are equipped with stronger ability to bind with Ca^2+^. Inspired by this, Kang et al. modified MPC polymers with ${\text{PO}}_{4}^{3-}$ to immobilize them on the tooth surfaces (Kang et al., 2016). Researches demonstrated that the introduction of ${\text{PO}}_{4}^{3-}$ ensured sufficient MPC coatings, resulting in increasing hydrophilicity and decreasing the adhesion of protein and *S. mutans*.

When the solution of bacteria or protein contacts the solid surface of materials, the new interaction between foulants and materials needs to be supplied to support the disruption of original liquid-liquid and solid-solid intermolecular force and to form the intermolecular force between the liquid and the solid surface. During this process, the former reflects the surface energy of liquid and solid materials respectively, and the latter represents the wettability of materials. So hydrophobic materials, or rather materials with lower surface energy, can weaken the interaction between foulants and materials, further improving the antiadhesion capability (Cazzaniga et al., 2015). The silicon-based materials are one of the most widely used polymeric antifouling agents for their low surface energy. Polysiloxane and its derivatives are typical silicone materials and have been demonstrated to serve as coatings for resistance to protein sorption (Yilgör and Yilgör, 2014; Santiago et al., 2016). Recently, Yu et al. synthesized a branched silicone methacrylate and incorporated it into the resin composites aiming at inhibiting the bacterial adhesion by decreasing the resin surface energy (Yu et al., 2020). Fluoropolymers are another option to serve as the low-surface-energy coatings for oral care. Churchley et al. synthesized a series of fluoropolymers and investigated their effectiveness as dental-care coatings (Churchley et al., 2008). These coatings behaved good resistance to several oral bacteria including *S. sanguinis*, *A. naeslundii* and cariogenic *S. mutans* and showed the potential of inhibiting acid demineralization. But it is regrettable that a correlation between anti-adhesion capability of fluoropolymers and their fluorine content or surface energy has not been established.

#### 3.1.2 Biomolecules

DNA is an emerging highly stable biopolymer in the biomedical field due to its unique and predictable properties. Attributed to the fact that some bacteria are known to deposit DNA to prevent the colonization of other bacteria around them, recent research has hypothesized that DNA coatings possess antifouling properties against bacteria (Berne et al., 2010). Subbiahdoss et al. coated DNA on the matrix by the layer by layer (LBL) technique to determine whether DNA coatings can inhibit microbial fouling (Subbiahdoss et al., 2019). Reduced number of several adherent bacteria on the DNA-coated matrix showed the potential in antifouling applications. Later, this team used a multilayer coating composed of DNA and chitosan by the LbL deposition on PMMA resins and Ti implants and demonstrated that these modified surfaces can prevent bacteria adhesion and biofilm formation (Ouni et al., 2021).

#### 3.1.3 Metal Oxide

The light-induced hydrophobic and hydrophilic transformation property of metal oxides enable them to be used for surface self-cleaning. Under the illumination of ultraviolet (UV) light with energy greater than the band gap, the valence band electrons of the metal oxides are excited to the conduction band, resulting in the formation of holes in the valence band. The holes “randomly walk” to the surface of the metal oxides and react with surface oxygen ions to form oxygen vacancies. At this time, oxygen vacancies can promote the dissociation and absorption of water molecules in the air to form a chemical adsorption surface (surface hydroxyl groups). Hydroxyl groups can further adsorb water molecules, thereby improving the hydrophilicity of the surface (Caputo et al., 2008; Sahoo et al., 2013). Since the viewpoint that the hydrophilicity and wettability of titanium dioxide (TiO_2_) polycrystalline films can be transformed by UV irradiation was proposed by Fujishima in 1997 (Wang et al., 1997), several metal oxides including ZnO, α-Fe_2_O_3_, WO_3_, V_2_O_5_ and SnO_2_ have been found successively to possess the photo-induced hydrophilicity (Feng et al., 2004; Lim et al., 2007; Papadopoulou et al., 2009; Gu et al., 2010; Talinungsang et al., 2019). For example, Papadopoulou et al. prepared ZnO nanograins by pulsed laser deposition and proved the light-induced superhydrophilicity in the hydrophobic structures (Papadopoulou et al., 2009). Yan et al. observed that the hydrophilic transformation behavior can also occur in α-Fe_2_O_3_ nanoflake films, whose contact angles can be switched from 160 to 0° upon stimulation brought from UV irradiation (Feng et al., 2004).

However, the light-induced hydrophobic and hydrophilic transformation property of metal oxides is reversible (Caputo et al., 2008). That is to say, the absorbed hydroxyl groups on the surface would be replaced again with oxygen in the air and hydrophilic materials return to their hydrophobic state once UV irradiation was stopped. The inevitable reversibility limits the application of metal oxides as light-induced antifouling materials in the treatment of oral diseases.

From the application point of view, the major disadvantage of metal oxides lies in its roughness and wettability, which is closely related to the surface topological structures (Packham, 2003; Chen et al., 2021). Hence, it is important to achieve their structure adjustable, especially at the micro/nanoscale. TiO_2_ nanomaterials are representative of nano-topological surfaces with the bacterial anti-adhesion property. Nowadays, nanostructured TiO_2_ materials with good wettability were extensively investigated in antifouling applications. Hu et al. constructed a composite nanostructure of TiO_2_ nanotubes on the substrates, which exhibited *S. sanguinis* and *S. mutans* antiadhesion behaviors (Hu et al., 2018). In addition to nanotubes, nanostructure surfaces such as nanopores, nanorods and nanogrooves have been indicated to possess good bacteria antiadhesion properties (Ferraris et al., 2017; Valdez-Salas et al., 2019). Besides, changing parameters of nanopatterns can lead to the changes of roughness and hydrophilicity on the surfaces, affecting the antiadhesion ability (Chen et al., 2021). For example, Krunal et al. studied the effects of different diameters of TiO_2_ nanotubes on the adherence of two oral bacteria S. *sanguinis* and *S. mutans* (Narendrakumar et al., 2015). In this study, they showed that the amount of attached bacteria can be adjusted as changing the nanotube diameters and demonstrated the possibility of tailoring nanostructure.

### 3.2 Antibacterial Materials

Antifouling agents have surely come into play in preventing microbial attachment and biofilm formation. Once bacteria are attached on the surfaces of teeth and dental materials to form the biofilm, antifouling agents are of no effect. While materials with antimicrobial properties are capable of killing those attached bacteria or destroying EPS according to several mechanisms and have become a strong candidate to regulate microbial environments.

#### 3.2.1 Metal and Metal Oxide

Many metal elements such as Ag, Cu, Zn and so on perform broad-spectrum antibacterial ability as positively charged metal ions (Ag^+^, Cu^2+^, Zn^2+^) can cause membrane destabilization and pore formation, leading to cytoplasmic metabolites leakage (Kędziora et al., 2018). However, releasing large amounts of metal ions in a short period of time will cause local excessive concentrations, producing a toxic effect on cells. To solve the problem, metal and metal oxide nanoparticles are used to achieve the ions’ slow release by the oxidative dissolution of ions from the nanoparticle surface. Besides, nanoparticles themselves have the ability of physical damage and membrane destabilization, which can reduce the number of needed metal ions (Gold et al., 2018). Dutra-Correa et al. functioned Ag nanoparticles with stabilizers to control the nanoparticle sizes and prevent aggregation (Dutra-Correa et al., 2018). These functioned nanoparticles can be incorporated into the dental adhesive at a lower concentration than that of previous studies. The antibacterial experiment and mechanical analysis demonstrated that Ag nanoparticles at a low concentration can still have the antibacterial effect on *S. mutans* without increasing the influence on mechanical properties of adhesive.

In addition to the contact-killing mechanism of metal nanoparticles, they can also lead to the change of surrounding environment such as elevating temperature or generating reactive oxygen species (ROS) to kill bacteria by response to external stimulus. Photothermal therapy (PTT) is a kind of emerging antibacterial means and has been achieved by the absorption of near-infrared light (NIR) of metal nanoparticles, especially Ag nanoparticles to generate heat, thus causing high temperature in the local to denature proteins of bacteria and kill them. Xu et al. developed a removable multilevel photothermal antibacterial nanoagent in which Fe_3_O_4_ was used as the core and polydopamine (PDA), Ag and glycol chitosan were coated in sequence (Xu et al., 2022). The existence of PDA slowed down the release of Ag^+^ so as to avoid tissue damage while the photothermal conversion property of Ag nanoparticles can realize effective sterilization within a short time when they were irradiated by NIR. The antibacterial experiment revealed the excellent bacterial and biofilm inhibition ratio (over 95% and 50% respectively) aiming at oral cariogenic bacteria.

**Figure 2:**
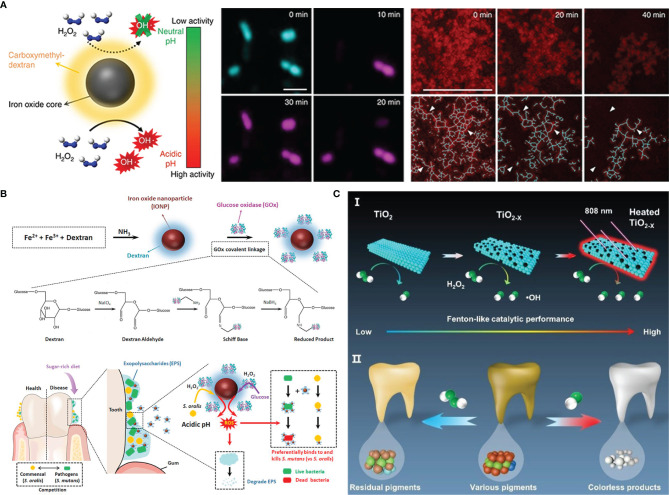
Metal nanoparticles antibacterial strategies: **(A)** combining Dex-IONP nanoparticles and additional H_2_O_2_ to generate ROS for disturbing biofilms; **(B)** Dex-IONP-GOx nanoparticles as nanoenzyme to release ROS for precision targeting of bacteria; **(C)** Oxygen-deficient nanotitania with enhanced photothermal Fenton-like reaction for destroying biofilms.

In 2007, paramagnetic Fe_3_O_4_ nanoparticles with the peroxidase-like activity were discovered by Yan’s team for the first time (Gao et al., 2007). The finding led to rapid development in the research for nanoparticles with similar property. So far, several kinds of metal and metal oxide nanoparticles including Fe_3_O_4_, Pt, Pd, Au, CeO_2_, CuO and so on have been confirmed to have the peroxidase-like activity (Fang et al., 2018; Xiang et al., 2020). Such nanoparticles with enzyme-like catalytic activity are also known as nanoenzyme, which can break down H_2_O_2_ to generate ROS at acidic pH values for degrading the biofilm EPS and simultaneously killing embedded bacteria. Gao et al. synthesized catalytic nanoparticles containing biocompatible Fe_3_O_4_ with peroxidase-like activity in a solvothermal system (Gao et al., 2016). These catalytic nanoparticles have been shown to activate exogenous H_2_O_2_ *in situ* to generate ROS that can achieve not only rapid bacteria killing but glucan degradation in biofilm EPS. Furthermore, the nanoparticles also exhibited an additional property of preventing hydroxyapatite demineralization, which was beneficial from the caries treatment. Likewise, Liu et al. designed a nanoparticles Ferumoxytol, which was comprised of iron oxide cores coated with carboxymethyl-dextran (Liu et al., 2018a). The nanoparticles also displayed biofilm disruption capability by activating H_2_O_2_ to cause *S. mutans* death and EPS matrix degradation (**Figure 2A**). The subsequent research revealed the antibacterial specificity of Ferumoxytol against *S. mutans*. They analyzed that the targeting property could be attributed to the interactions between carboxymethyl-dextran of Ferumoxytol and specific glucan-binding proteins of *S. mutans* (**Figure 2B**) (Liu et al., 2021b). Although these catalytic nanoparticles exhibited excellent antibacterial properties, the inappropriate additive amount of exogenous H_2_O_2_ can induce excess ROS causing cell damage. Considering that, glucose oxidase (GOx), an endogenous oxidoreductase that can catalyze the oxidation of β-D glucose into H_2_O_2_, has come into view (Chaichi and Ehsani, 2016; Laothanachareon et al., 2018). Ji et al. prepared Fe_3_O_4_ nanoparticles and modified them with GOx (Ji et al., 2021). GOx can catalyze glucose in the biofilm matrix to generate H_2_O_2_, which can be further catalyzed by Fe_3_O_4_ nanoparticles to produce ROS. In addition, the oxidation of GOx depleted the oxygen and glucan, helping to starve bacteria to death. Inspired by the specific binding of glucan and oxidation capability of GOx, Huang et al. coated iron oxide nanoparticles with both glucan and GOx (Huang et al., 2021). The resulting nanohybrid had significant *S. mutans* killing efficacy without affecting commensal *S. oralis*. To reduce the used amount of nanoparticles, these metals can be fixed in other materials such as carbon nitride and metal-organic frameworks (MOF) (Wang et al., 2020; Wu et al., 2021b). Yu et al. reported single-atom dopped MOF catalytic systems with several metal atoms including Pt, Au, Cu, and Ru for the treatment of periodontitis (Yu et al., 2022). Due to its three-dimensional and porous structure, the MOF-based catalytic system had plentiful catalytic sites so as to improve catalytic activity and reduce metal consumption (Liu et al., 2020).

In addition to being a light-responsive antifouling agent, TiO_2_ is a commonly used photocatalyst, which could respond to UV to generate ROS for destroying microbes. However, exposure to UV light is harmful to cells and tissues restricts the application of TiO_2_ (Musk et al., 1989). So, the current advancements mainly focus on facilitating visible light adsorption by narrowing the TiO_2_ band gap (Asahi et al., 2001). Previous literature found that doping with nitrogen endowed TiO_2_ with superior visible light-catalytic activity (Livraghi et al., 2006). Inspired by this, Florez et al. synthesized nitrogen-dopped TiO_2_ nanoparticles and immobilized them in the dental adhesive resins (Esteban Florez et al., 2018). There was a higher antibacterial level when exposed to blue light than in the dark, which demonstrated the contribution of nitrogen to band-gap narrows. In addition to nonmetal atoms doping, oxygen-deficient titania (TiO_2-x_) can exhibit better photo-catalytic performance than TiO_2_ due to its improved separation of electron-hole pairs and extended visible light absorbance regions (Chen et al., 2011; Naldoni et al., 2012). Hu et al. prepared TiO_2-x_ nanoparticles from TiO_2_ based on the solid-state chemical reduction method (Hu et al., 2021). The presence of oxygen vacancy improved the catalytic activity under NIR irradiation and meanwhile, elevated temperature brought from photo-thermal conversion can also kill bacteria (**Figure 2C**).

#### 3.2.2 Inorganic Nonmetallic Materials

As the two main inorganic nonmetal materials, carbon nanotube and graphene oxide (GO) have a similar antibacterial mechanism. The penetration of the sharp and narrow structure of two materials onto the surface of bacteria can cause damage in the integrity of cell walls (Teh and Lai, 2019). F. Al-Thani et al. have studied the antibacterial efficiency of GO and concluded that GO can work against several microbiomes including eukaryotic fungus, Gram-negative and positive bacteria (Al-thani et al., 2014).

Nitric oxide (NO) is an endogenous diatomic radical whose antibacterial activity origin from its reaction with superoxide and oxygen. In the process, peroxynitrite and dinitrogen trioxide were formed to kill bacteria through lipid peroxidation and DNA cleavage (Bogdan, 2001; Hetrick et al., 2008). Compared with direct delivery of NO, a NO-releasing system will be applicable to oral surgery. J. Backlund et al. loaded NO into PAMAM dendrimers and discussed the influence of different pH and the alkyl chains length of dendrimers on NO-release kinetics (Backlund et al., 2016). Improved antibacterial actions can be observed at lower pH values and when NO was loaded into longer alkyl chain-modified dendrimers. Similarly, NO-releasing hyperbranched polykanamycins and hyperbranched polyamidoamines systems designed by Yang et al. can not only reduce the metabolic activity of biofilm, but also kill embed bacteria. The greater efficacy was observed under aerobic versus anaerobic conditions (Ma et al., 2020).

#### 3.2.3 Organic Small Molecules

The gold standard aiming at oral bacteria in the clinical treatment is the use of antibacterial agent chlorhexidine (Balagopal and Arjunkumar, 2013). Recent advances about the delivery of chlorhexidine in different carrier systems can achieve a slow release or controlled release of chlorhexidine for prolonging the releasing time and reducing drugs usage. Akram et al. reported a strategy that mesoporous silica nanoparticles (MSNs) were grafted with poly (L-glycolic acid) to load chlorhexidine and studied the release behaviors under the oral acid-producing environment (Akram et al., 2021). Equipped with exceptional surface area and porous structures, MSNs can load drugs for improving efficacy. PGA is a kind of synthesized polypeptide with the pH-responsive property, which guaranteed a significant effect on chlorhexidine release behaviors and nanoparticles degradation.

Quaternary ammonium salts (QAS) have been one of the most widely studied antibacterial agents on account of their chemical structure with ease of design and modification. The antibacterial capability originates from the interactions between cationic QAS molecules and the bacterial cell membranes with negative charges (Ramburrun et al., 2021). The antibacterial property of QAS can be optimized by changing the length of alkyl chains of QAS molecules. For example, QAS molecules with longer alkyl chains (C6-C18) are more applicable to kill bacteria because long alkyl chains can disrupt the phospholipid molecules on the cell membranes (Jiao et al., 2017). While relating to the antifungal therapy, it’s necessary to expose more quaternary ammonium groups with positive charge, thus shorter alkyl chains are rather needed (De Prijck et al., 2010; Duan et al., 2022). Dimethylaminohexadecyl methacrylate (DMAHDM) is a kind of QAS antibacterial monomer with an alkyl length of 16, showing strong antibacterial activity and antibacterial efficacy (Zhou et al., 2013). Bhadila et al. developed a bioactive antibacterial composite with DMAHDM and amorphous calcium phosphate (ACP) (Bhadila et al., 2020). The composite can not only protect dentin at the restoration margins from invading of *S. mutans* biofilm, but also promote dentin remineralization. As a small molecular antibacterial agent, QAS can also be grafted onto polymer chains with biological functions to play an antibacterial role. Fanfoni et al. designed and synthesized a series of di-methacrylate bis-QAS that bear two quaternary ammonium groups in a monomer (Fanfoni et al., 2021). These synthesized monomers had the potential of stabilizing polymer networks as crosslinkers, and the existence of two quaternary ammonium groups increased the antibacterial activity.

**Figure 3:**
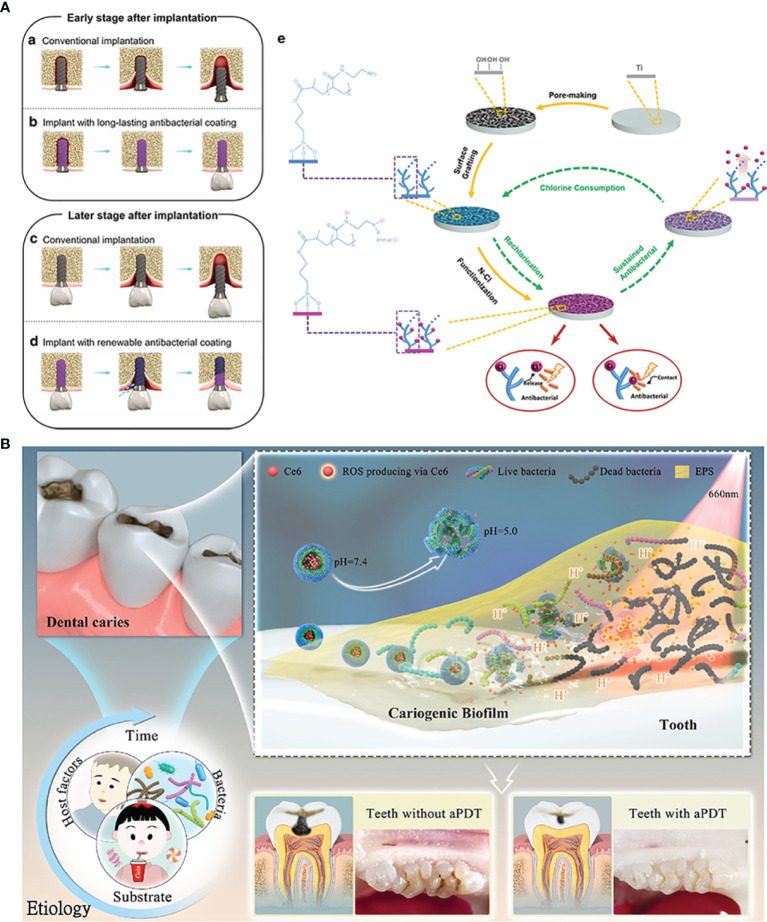
Organic micromolecules antibacterial strategies: **(A)** the renewal of active chlorine from N-halamines coating to achieve long-lasting antibacterial property; **(B)** chlorin e6-mediated PDT therapy for bioresponsive bacterial resistance.

N-halamines are a class of small molecular compounds with one or several nitrogen-halogen (N-X) bonds, in which X could be Cl, Br or I. Among them, Cl is the most widely used element because of the most advantageous stability of N-Cl bonds. The antibacterial property of N-halamines originates from the release of Cl^+^. Releasing Cl^+^ first chlorinates the external protein matrix of the bacteria to form a protective layer around the bacteria, which helps it penetrate into the bacterial cells. Cl^+^ going into the bacteria further oxidizes the key cellular components containing mercaptan and sulfide, and finally denatures the proteins by counter-chlorination (Dong et al., 2017). It can be seen from the structure of N-halamines that the dissociation constant of Cl element in aqueous solution decreases in the order of imide > amide > amine, which means that the Cl^+^ releasing capability decreases in the same order (Akdag et al., 2006). Contrarily, the durable stability of N-halamines can get improved in order. Wu et al. grafted polyacrylic acid (PAA) onto Ti implants for N-Cl functionalization to acquire porous renewable antibacterial coatings (Wu et al., 2021a). In the research, they utilize excess ethanediamine to react with PAA to ensure that the resulting coatings can contain not only amide but also amine. Such molecular design can provide a synergistic antibacterial effect of rapid and long-lasting functions (**Figure 3A**).

Antibacterial photodynamic therapy (PDT) enjoys a tough interest in current oral and dental applications. Photosensitizers around tissues are activated by the light irradiation of a specific wavelength, and the excited photosensitizers transfer the energy to the surrounding oxygen to generate the highly active ROS, which can oxidize the adjacent biological macromolecules for killing bacteria (Stájer et al., 2020). Current photosensitizers used for PDT are porphyrin, chlorophyll, toluidine blue O (TBO), phthalocyanine compounds and these derivatives (Li et al., 2021). Zhang et al. designed a zwitterion-modified porphyrin by the conjugation of protoporphyrin IX (PP) and a zwitterion moiety (Zhang et al., 2021). PP segments can improve the generation of ROS by purple light irradiation for tooth whitening and *S. mutans* biofilm eradication, while the superhydrophilic zwitterion can increase the solubility of modified porphyrin and ROS yields. Chlorin e6 (Ce 6), a class of small molecular photosensitizer extracted from natural chlorophyll, has been revealed to have a brilliant ROS generation efficacy and absorption of visible red light (Ding et al., 2018). Liu et al. once designed an amphiphilic and pH-responsive polymer, which can self-assemble into spherical structure in a neutral condition and disassemble under an acidic environment. Considering the lower pH in the caries environment, they encapsulated Ce 6 with the polymer for PDT on demand (**Figure 3B**) (Liu et al., 2021a). A. Balhaddad et al. constructed a nanoplatform by assembling TBO and magnetic Fe_3_O_4_. In addition to the photodynamic antibacterial property of Ce 6, Fe_3_O_4_ equipped the nanoplatform with the capability to penetrate deep sites under external magnetic forces, resulting in an improved disinfection effect (Balhaddad et al., 2021).

#### 3.2.4 Polymers

Compared to inorganic and organic small molecular antimicrobial agents, polymer with antibacterial activity is a hot topic of current research due to its high density of effective functional groups. Usual polymers can be divided into synthetic polymers and natural polymers. Among them, Polyethyleneimine (PEI) is a typical synthetic cationic antibacterial polymer, which can interact with the polar acid groups on the bacteria to destroy cell membranes (Pietrokovski et al., 2016). Karatepe et al. incorporated PEI and silk fibroin (SF) into dental resins. In addition to reinforced mechanical strength brought from SF, PEI endowed resins with the resistance to bacterial erosion of *P. aeruginosa* (Karatepe and Ozdemir, 2020).

As extracted from matters in nature, natural polymers often exhibit low toxicity, good biocompatibility and biodegradation. For example, chitosan is extracted by the deacetylation of chitin and the positively charged ammonium groups ( ${\text{NH}}_{3}^{+}$ ) can be generated upon protonation of amino groups. ${\text{NH}}_{3}^{+}$ can interact with negatively charged bacterial cell membranes to cause leakage (Benhabiles et al., 2012). Peng et al. reported an antimicrobial coating by incorporating PEG and chitosan to combat bacterial infection (Peng et al., 2020). Herein, the coating showed a long-lasting colony-suppression activity against *S. mutans*. Similar to QAS, the length of hydrophobic groups can also influence the antibacterial activity. Phuangkaew et al. introduced hydrophobic entities and quaternary ammonium groups to improve the antibacterial capability (Phuangkaew et al., 2022).

#### 3.2.5 Antimicrobial Peptides

Natural AMPs are a class of polypeptides with broad antibacterial activity extracted from plants, amphibians or human bodies, which are usually composed of hydrophobic regions and positively charged hydrophilic regions (Parhi et al., 2021). The hydrophobic regions, such as tryptophan and leucine, can be in combination with the phospholipid bilayer membrane, while the presence of hydrophilic positively charged arginine and lysine can play an antibacterial role. Even though human oral saliva contains different kinds of AMPs, when acting on oral microorganisms, the minimum inhibitory concentration (MIC) should be reached. It is worth noting that the concentration of natural AMPs in the gingival crevicular fluid is much lower than MIC of most microorganisms. Although oral endogenous AMPs are not enough to produce antibacterial effect on pathogenic bacteria, the wide range of sources provides a new idea for the treatment of oral diseases with additional AMPs. A variety of AMPs, including α-defensin, β-defensin, histatin, and histoprotestatin (such as LL-37), are normally present in oral saliva and have been shown to have antibacterial effects against multiple oral bacteria (Gorr, 2009).

The antibacterial activities of natural AMPs have been extensively studied, but their sources are limited and polypeptide chains are too long and complex to be flexible. On the contrary, *de novo* designed AMPs and the antibacterial units extracted from natural AMPs can solve these problems without impairing the antibacterial activity. For example, G(IIKK)_3_I-NH_2_ (called as G3) is a man-made helical peptide, and has been proven to have antibacterial activity against *S. mutans* biofilms (Zhang et al., 2020). P-113 (AKRHHGYKRKFH-NH_2_) is a histidine-rich 12-amino acid polypeptide from saliva protein histatin 5. In light of previous research, P-113 has bactericidal effects on oral important pathogenic microorganisms (Rothstein et al., 2001; Sajjan et al., 2001). Wang et al. provided a novel and stable Nal-P-113 by replacing tryptophan and histidine residues with the bulky amino acids β-naphthylalanine and β-(4,4’-biphenyl) alanine to increase salt resistance. The variant AMP retained high antibacterial activity against *Stoeptococcus gordonii*, *F. nucleatum* and *P. gingivalis* even at high salt concentrations (Wang et al., 2015).

### 3.3 Materials for the Disease Treatment

**Figure 4:**
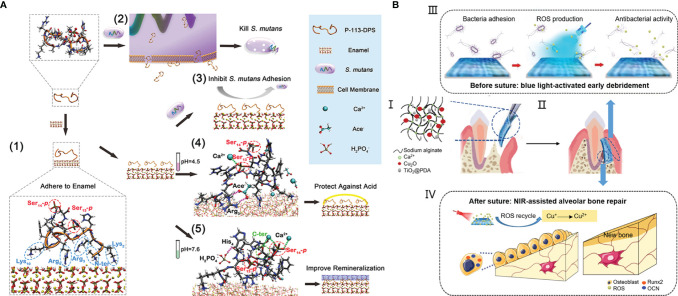
The combined treatment of oral diseases: **(A)** a multifunctional antibacterial peptide coating with modified end groups for adhering to enamel, bacterial anti-adherence and enamel remineralization to achieve caries management; **(B)** an injectable dual light-responsive GTR membrane with the antibacterial property and osteogenic capability to address requirements of periodontitis therapy.

The emergence of antifouling and bactericidal materials provides a new means for the treatment of oral diseases caused by dysbiosis of bacteria. However, the actual oral environment determines the diversity of causes and complexity of results of oral diseases, so single anti-fouling or bactericidal performance is not enough to meet the needs of disease treatment. For example, the overgrowth of oral caries-causing bacteria *S. mutans* is the direct cause of dental caries. In this process, the local pH of oral cavity is also decreased, which further leads to tooth hard tissue demineralization in acidic environment. Consequently, the treatment for dental caries is usually involving a combination of antifouling/antibacterial property and promoting tooth remineralization. Zhou et al. grafted P-113 (the smallest fragment of AMP H5) with different end moieties in order to achieve binding to tooth enamel, killing *S. mutans*, resisting demineralization and promoting remineralization (Zhou et al., 2021). The study suggested the potential of modified P-113 as the functional agent for preventing dental caries (**Figure 4A**). To inhibit the failure of resin-based dental materials brought from recurrent caries, Melo et al. filled resin with Ag nanoparticles, DMAHDM and ACP. In addition to the antibacterial activity of Ag and DMADHM, ACP can release Ca^2+^ and ${\text{PO}}_{4}^{3-}$ for remineralization and acid neutralization (Melo et al., 2016).

As one of the most common chronic infections, periodontitis will result in the destruction of periodontal tissue including alveolar bone, periodontal ligament and cementum root. The ultimate goal of periodontal therapy is the regeneration of all periodontal components, while the therapy usually combines conventional anti-infective measures with guided tissue regeneration (GTR) or the application of cytokines, growth factors, or bioactive molecules (Bottino and Thomas, 2015). Nasajpour et al. developed a biodegradable GTR membrane made with a mixed solution of poly(caprolactone) and ZnO by electrospinning for treating periodontitis (Nasajpour et al., 2018). The incorporation of ZnO improved the antibacterial activity and osteoconductivity simultaneously. Xu et al. proposed an injectable sodium alginate hydrogel containing Cu_2_O and PDA-coated TiO_2_ (Xu et al., 2020). The liquid to solid phase transition during the gelation process can make the hydrogel match the irregular defect sites. The blue light-responsive property of TiO_2_ can generate ROS that can not only kill bacteria but also oxide Cu^+^ to Cu^2+^ for stimulating osteogenesis (**Figure 4B**). Zhang et al. developed a microneedle patch for drug delivery of antibiotics and cytokines IL-4 and TGF-β to achieve immunoregulation and tissue regeneration (Zhang et al., 2022).

Like periodontal infection, peri-implantitis is a multimicrobial disease that causes bone absorption and ultimately implant failure. In view of the fact that bacterial infection is the main cause of peri-implantitis, the common treatment method is still to improve the antibacterial performance of implants through bacterial adhesion prevention and sterilization (De Avila et al., 2020). At present, most implants are made of pure titanium and titanium alloy materials. However, titanium implants widely used in clinical practice do not have outstanding anti-infection ability (Chen et al., 2021). More recently, researchers have tried to kill bacteria by mixing pure titanium or its alloys with other metals such as Ag, Cu, and Zn that have inherent antibacterial properties (Chen et al., 2016; Wang et al., 2019). Another way to improve the antibacterial ability of implants is the usage of an antifouling or bactericidal material as the coating of the implant surface. Hoyos-Nogués et al. presented a three-in-one trifunctional strategy by preparing a coating with PEG, AMP and RGD tripeptide. The strategy can promote the attachment and spreading of osteoblasts on implant surfaces and inhibit bacterial colonization on them (Hoyos-Nogues et al., 2018).

## 4 Conclusions and Perspectives

The oral ecosystem contains several distinct niches, which support the colonization of complex and heterogeneous microbial communities. There are dynamic interactions between oral environments and the compositions of oral microbiota and between oral microorganisms. These interactions can prevent humans from invasion and attack. The oral microbiome is individual and relatively stable as time goes on as long as the oral health is maintained. However, the significant change of key parameters influencing microbial growth will disturb the balanced interactions and lead to the development of pathogenic microorganisms. Once the oral microbial dysbiosis occurs, people are susceptible to being attacked by oral diseases such as dental caries, periodontitis, and peri-implantitis.

There is a close relationship between the occurrence of oral diseases and the overgrowth of pathogenic bacteria and the formation of their biofilms. In the past few decades, the development of materials science, chemistry and biomedical engineering as well as their intersection promote the blooming research aiming at antibacterial materials. The methods of resisting microbial invasion involve antiadhesion, sterilization and even their combination. In addition to hydrophilic and hydrophobic materials that have been studied extensively, bioinspired DNA is an optional antifouling agent. The photoinduced hydrophobic-hydrophilic transformation property of TiO_2_ and its modification of surface morphology can achieve controllable bacterial adhesion, showing potential in preventing peri-implantitis. There is a wider range of bactericidal materials, ranging from inorganic materials such as metals and carbides to organic small molecules, synthetic polymers and some natural molecules. the development of distinct categories of materials enriches antibacterial means: metal ions, chlorhexidine and QAS are still mainstreams, while the application of NO, Cl^+^ and AMP is also increasingly emerging. Moreover, mature nanotechnology makes it possible for nanoenzyme, PTT and PDT to be used in the treatment of oral diseases, which further expands the application of some metal and organic molecules. These antibacterial materials have been combined with other methods for the research of treating oral diseases such as dental caries, periodontitis and peri-implantitis based on the characteristics of different oral diseases, showing excellent results.

Although the research on oral antibacterial materials is thriving, these materials are not widely used in clinic. The antibacterial experiment *in vitro* only focuses on one or several pathogenic bacteria. Considering the complexity of microorganisms in the oral environment, it is difficult to predict the effect of antibacterial materials applied to oral cavity. Materials possessing bactericidal effects usually have cytotoxicity as well. In order to achieve good antibacterial properties, it is usually necessary to increase the concentration of materials with low antibacterial activity, which may cause worse biocompatibility. Therefore, a balance between the antibacterial activity and biocompatibility of materials needs to be found in the future. Finally, the results of basic research should be effectively translated into real and affordable products, which requires the joint cooperation and efforts of researchers, doctors and patients.

## Author Contributions

JZ and WC drafted the manuscript. JLuo and JLi provided valuable insights for the manuscript. JY and LH reviewed and edited the manuscript. All authors have approved the final version of the manuscript.

## Funding

This work was supported by National Natural Science Foundation of China (51903169, 81991500, 81991501 and 82170949); Key Research and Development Program of Sichuan Province (2021YFS0057 and 2020YFS0180).

## Conflict of Interest

The authors declare that the research was conducted in the absence of any commercial or financial relationships that could be construed as a potential conflict of interest.

## Publisher’s Note

All claims expressed in this article are solely those of the authors and do not necessarily represent those of their affiliated organizations, or those of the publisher, the editors and the reviewers. Any product that may be evaluated in this article, or claim that may be made by its manufacturer, is not guaranteed or endorsed by the publisher.

## Abbreviations

EPS, extracellular polymeric substances; ADS, arginine deiminase system; IPS, intracellular polysaccharides; PRRs, pattern recognition receptors; PAMP, pathogen-associated molecular patterns; IL-1, interleukin-1; IL-6, interleukin-6, TNF, tumor necrosis factor; TLRs, toll-like receptors; IL-8, interleukin-8; Th17, T helper 17 cells; IL-17, interleukin-17; IL-23, interleukin-23; TGF-β, transforming growth factor-β; MMP, matrix metalloproteinases; PEG, polyethylene glycol; MPC, 2-methacryloyloxyethyl phosphorylcholine; Ti, titanium; PEG-PAsp, polyethylene glycol-poly (aspartic acid); LBL, layer by layer; TiO_2_, titanium dioxide; UV, ultraviolet; ROS, reactive oxygen species; PTT, Photothermal therapy; NIR, near-infrared light; PDA, polydopamine; GOx, glucose oxidase; MOF, metal-organic frameworks; TiO_2-x_, oxygen-deficient titania; GO, graphene oxide; NO, nitric oxide; MSNs, mesoporous silica nanoparticles; QAS, quaternary ammonium salts; DMAHDM, dimethylaminohexadecyl methacrylate; ACP, amorphous calcium phosphate; N-X, nitrogen-halogen; PAA, polyacrylic acid; PDT, photodynamic therapy; TBO, toluidine blue O; PP, protoporphyrin IX; Ce 6, Chlorin e6; PEI, polyethyleneimine; SF, silk fibroin; ${\text{NH}}_{3}^{+}$ , positively charged ammonium groups AMPs, antimicrobial peptides; MIC, minimum inhibitory concentration; GTR, guided tissue regeneration.
